# Malignant sacrococcygeal germ cell tumors in children in Taiwan: A retrospective single-center case series

**DOI:** 10.1097/MD.0000000000024323

**Published:** 2021-01-29

**Authors:** Shih-Hsiang Chen, Chia-Jui Du, Jin-Yao Lai, Tsung-Yen Chang, Chao-Ping Yang, Iou-Jih Hung, Tang-Her Jaing, Yung-Ching Ming, Chuen Hsueh

**Affiliations:** a Division of Pediatric Hematology-Oncology; b Division of Pediatric General Medicine; c Division of Pediatric Surgery; d Department of Pathology, Chang Gung Memorial Hospital, College of Medicine, Chang Gung University, Taoyuan, Taiwan.

**Keywords:** children, malignant sacrococcygeal germ cell tumors, outcomes, teratoma, yolk sac tumor

## Abstract

Although the incidence of malignant sacrococcygeal germ cell tumors (MSGCTs) is high in the East Asian countries, information about MSGCTs from this region is limited. This report aimed to analyze the data of children with MSGCTs in a single medical center in Taiwan.

Patients aged 18 years or younger with primary MSGCTs or malignant recurrence of a sacrococcygeal teratoma who underwent surgery during the neonatal period between January 1999 and December 2016 were identified from the Linkou Chang Gung Cancer Center registry. The clinical features, laboratory data, and treatment outcomes were reviewed.

Fifteen children (1 man and 15 women) with MSGCTs were identified. Sacrococcygeal tumors were present at birth in 7 patients. All patients presented with a bulging mass at the buttock region and they had normal alpha-fetoprotein levels at the time of diagnosis. They underwent primary excision of the tumor. Immature teratoma was histologically diagnosed in 5 neonates, and mature teratoma in 2. Only 1 patient with grade 3 immature teratoma received adjuvant chemotherapy. Two patients with mature teratoma developed malignant recurrence 1.6 and 2.1 years later, respectively. Eight patients were diagnosed with MSGCTs after the neonatal period. The common presenting symptoms included buttock asymmetry (37.5%), abdominal distension (25%), and constipation (12.5%). Seven patients had elevated alpha-fetoprotein levels for their age. They were administered neoadjuvant chemotherapy followed by tumor excision if a residual tumor was present. The histology of the excised tumor included mature teratoma (66.7%) and necrosis (33.3%). One patient with a normal alpha-fetoprotein level underwent primary tumor excision followed by adjuvant chemotherapy. Grade 2 immature teratoma with embryonal carcinoma was diagnosed histologically. Among the 15 patients with MSGCTs, 3 had a recurrence (at age of 2.1, 0.5, and 2.4 years, respectively) and 1 died (at age of 6.1 years) of disease progression. The 5-year overall and event-free survival rates were 90% and 80%, respectively.

Children with MSGCTs had good overall prognoses in this case series. For those with sacrococcygeal mature teratoma or low-grade immature teratoma in the neonatal period, we recommend close follow-up for at least 3 years after surgery to detect malignant recurrence.

## 1 Introduction

Malignant germ cell tumors constitute approximately 3% to 4% of all malignancies in children.^[1]^ The sacrococcygeal region is the most frequent extragonadal localization of germ cell tumors in infancy and childhood.^[2]^ Mature and immature teratomas account for the majority of sacrococcygeal germ cell tumors in infancy, whereas the yolk sac tumor represents the most common histology type in children beyond the neonatal period. Mature teratomas and low-grade immature teratomas are usually treated by gross complete resection. However, malignant germ cell tumors might recur at the same location after resection of the sacrococcygeal teratoma.^[3]^ It has been reported that the incidence of germ cell tumors is very high in some Asian countries including Taiwan than in Europe, the United States, Australia, China, and Japan.^[4]^ However, previous reports from Korea and Taiwan focused on the treatment outcome of all extracranial malignant germ cell tumors.^[5,6]^

Hou et al^[5]^ analyzed 81 children with extracranial germ cell tumors in Taiwan. Complete surgical excision when feasible followed by adjuvant chemotherapy was the major concept of treatment. For those with unresectable tumors at diagnosis, a biopsy was performed first. They underwent neoadjuvant chemotherapy followed by second-look surgery. The 10-year overall survival and event-free survival rates of the entire cohort were 95% and 88%, respectively. Kim et al^[6]^ reported the treatment outcome of 66 Korean children with extracranial germ cell tumors. The 5-year overall survival and event-free survival rates were 92% and 90%, respectively. Malignant sacrococcygeal germ cell tumors (MSGCTs) accounted for 6% of extracranial malignant germ cell tumors in both reports. Limited information about children with MSGCTs from these countries prompted us to conduct this study to analyze the data of children with MSGCTs in a single medical center in Taiwan.

## 2 Methods

Patients aged 18 years or younger with primary MSGCTs or malignant recurrence of a sacrococcygeal teratoma operated during the neonatal period were identified from the Linkou Chang Gung Cancer Center registry. Mature teratoma was not included in this study. The study was approved by the Institutional Review Board of Linkou Chang Gung Memorial Hospital. The clinical features, laboratory data, and treatment outcomes were retrospectively reviewed from the medical records.

The initial diagnosis was based on the clinical and radiological evidence of a sacrococcygeal tumor and serum alpha-fetoprotein (AFP) level. The investigation of metastasis included computed tomography of the chest and abdomen, and bone scan. The histopathological diagnosis and classification of germ cell tumors were made by the pathologists with a combination of histopathology and immunohistochemistry mostly in accordance with the modern World Health Organization classification published in that era. Briefly and generally, mature teratoma contains well-differentiated elements of at least 2 germ cell layers (endoderm, ectoderm, and/or mesoderm). Immature teratoma is incompletely differentiated and is similar to fetal or embryonic tissue. Grading of immature teratoma is based on the amount of immature neuroepithelium: 1 low-power field in any one slide (grade I), 1–3 low-power fields in any one slide (grade II), exceeds 3 low-power fields in any one slide (grade III). Yolk sac tumor is characterized by sheets of tumor cells with prominent cytoplasmic vacuoles exhibiting specific patterns such as reticular pattern, endodermal sinus pattern, or others.

The treatment strategy was modified from the Children's Oncology Group (previously the Children's Cancer Group [CCG] and the Pediatric Oncology Group [POG]) protocol.^[7]^ In general, the primary excision of the sacrococcygeal tumor was performed in neonates with operable tumors and normal serum AFP levels for age. A posterior transacral incision with removal of the coccyx is the standard approach in our institution. Four to 6 cycles of adjuvant chemotherapy were administered in patients with grade III immature teratoma or the presence of a malignant component. Children beyond the neonatal period with evidence suggestive of malignancy (i.e., high AFP level for age) received neoadjuvant chemotherapy followed by delayed tumor excision. Six cycles of chemotherapy were applied to most patients. After 4 cycles of chemotherapy, patients with evidence of gross residual tumor underwent excision of the sacrococcygeal tumor. The regimen of chemotherapy was BEP (cisplatin 20 mg/m^2^/d on days 1–5, etoposide 100 mg/m^2^/d on days 1–5, and bleomycin 15 mg/m^2^ on day 2) or JEB (carboplatin 600 mg/m^2^ on day 1, etoposide 120 mg/m^2^/d on days 1–3, and bleomycin 15 mg/m^2^ on day 2) in 3-week intervals.

After completion of treatment, all patients were followed by clinical examination and AFP measurement every 1 to 2 months for the first year, every 3 to 4 months for the second year, every 4 to 6 months for the third to fifth years, and annually thereafter. Image studies were performed when necessary. Overall survival (OS) was measured from the time of diagnosis of primary MSGCTs or malignant recurrence of a sacrococcygeal teratoma to the date of death, or until the last follow-up in patients who were alive. Event-free survival (EFS) was measured from the time of diagnosis of primary MSGCTs or malignant recurrence of a sacrococcygeal teratoma to the date of event in patients who experienced an event, or if no failure was observed, until the last follow-up. The events considered were recurrence, disease progression, or death, whichever occurred first. Recurrence was defined as re-emergence of serum AFP level in previous negative or declining level, and/or the radiological evidence of recurrent tumor locally or distantly after completion of treatment. Disease progression was defined as persistent or increasing serum AFP level, progressive enlargement of the local tumor, and/or the occurrence of distant metastasis under treatment. Kaplan–Meier analysis was used to evaluate survival.

## 3 Results

Between January 1999 and December 2016, 15 children with MSGCTs were identified. The median age at the time of diagnosis of MSGCT was 1.3 years (range, 9 days–8.1 years). Of the 15 children, 1 was man and 14 were women.

Seven patients were diagnosed with a sacrococcygeal germ cell tumor in the neonatal period (Table 1). Of these, 5 had primary MSGCTs and 2 had a malignant recurrence of a neonatal sacrococcygeal teratoma. All 7 patients were women and presented with an external sacral mass. Tumors in 4 patients were prenatally detected by fetal ultrasonography, and these patients were delivered by cesarean section. Among the remaining 3 patients, 2 were delivered by cesarean section and 1 by vaginal birth. No significant comorbidities and complications were observed during birth in all 7 patients. None had metastatic lesions at the time of diagnosis of the neonatal sacrococcygeal tumor. The serum AFP level of the 5 patients with primary MSGCT ranged from 28,600 to 312,177 ng/mL (within the normal range for age). The primary excision of the sacrococcygeal tumor was performed in all 7 patients. Immature teratoma, immature teratoma with yolk sac component, and mature teratoma were histologically diagnosed in 4 (57%), 1 (14%), and 2 (29%), respectively. Adjuvant chemotherapy was administered in 2 patients; 1 had grade III immature teratoma and 1 had grade II immature teratoma with yolk sac component. In the 2 patients with mature teratoma, malignant recurrence occurred 1.6 and 2.1 years after the primary diagnosis.

**Table 1:** Clinical characteristics and outcome of 7 patients with neonatal sacrococcygeal germ cell tumors.

Case	Age at diagnosis	Sex	Prenatal detection	Delivery type	AFP at diagnosis	Metastasis	Surgery	Histology	Chemotherapy	Outcome
Malignant recurrence of a sacrococcygeal teratoma										
1	27 days	F	No	C/S	7,431	No	Total excision	MT	No	Recurrence (YST, 2.1 years later)
^¶^1a	2.1 years				330,181	No	^∗^Total excision	^†^MT	^‡^JEB × 9	Recurrence, dead
2	23 days	F	No	C/S	55,884	No	Total excision	MT	No	Recurrence (YST, 1.6 years later)
^¶^2a	1.6 years				153.6	No	Total excision	YST	^§^JEB × 4	DFS
Primary malignant sacrococcygeal germ cell tumor										
3	19 days	F	Yes	C/s	28,600	No	Total excision	Grade I IT	No	DFS
4	22 days	F	No	NSD	28,800	No	Total excision	Grade II IT	No	Recurrence (IT, 0.4 years later), DFS
5	10 days	F	Yes	C/S	312,177	No	Total excision	Grade III IT	^§^JEB × 6	DFS
6	14 days	F	Yes	C/S	263,078	No	Total excision	Grade II IT	No	DFS
7	9 days	F	Yes	C/S	150,782	No	Total excision	Grade II IT + YST	^§^JEB × 4	DFS

BEP = bleomycin, etoposide, and cisplatin; C/S = cesarean section; DFS = disease-free survival; IT = immature teratoma; JEB = carboplatin, etoposide, and bleomycin; MT = mature teratoma; NSD = normal spontaneous delivery; YST = yolk sac tumor.

∗ Total excision was done after 4 cycles of neoadjuvant chemotherapy.

† The histology examination was done after neoadjuvant chemotherapy.

‡ The chemotherapy comprised 4 cycles of neonadjuvant chemotherapy and 5 cycles of adjuvant chemotherapy

§ Adjuvant chemotherapy

¶ Malignant recurrence of a neonatal sacrococcygeal teratoma

Eight patients were diagnosed after the neonatal period (Table 2). All were women except one. The median age at the time of diagnosis was 1.7 years (range, 0.4–8.1 years). The common presenting symptoms included buttock asymmetry (37.5%), abdominal distension (25%), and constipation (12.5%). Serum AFP level was high in 7 patients (range, 40,300–453,173 ng/mL), while 1 patient presented with normal AFP level at diagnosis. Six patients had metastatic lesions at diagnosis, and the liver was the most common metastatic site. Four patients underwent a biopsy. Yolk sac tumor was confirmed in 3 (75%), and 1 had immature teratoma (25%). Six patients received neoadjuvant chemotherapy followed by surgical excision. One patient had no residual tumor after neoadjuvant chemotherapy. The patient who had a normal serum AFP level and was diagnosed with immature teratoma on biopsy underwent primary tumor excision first. Adjuvant chemotherapy was administered due to the presence of a malignant component in the resected surgical specimens.

**Table 2:** Clinical characteristics and outcome of 8 patients with sacrococcygeal germ cell tumors diagnosed after neonatal period.

Case	Age at diagnosis	Sex	Symptoms	Initial AFP	Metastasis	Biopsy	NACT	Posttherapy surgery	^∗^Histology	ACT	Outcome
8	1.8 years	M	Abdominal distension	126,500	Liver	No	BEP × 6	No	No	No	DFS
9	1.6 years	F	Constipation	249,000	Liver, Lung	Yes (YST)	BEP × 4	Total excision	Necrosis	BEP × 2	DFS
10	1.3 years	F	Buttock asymmetry	453,173	Liver, bone	Yes (YST)	BEP × 4	Total excision	MT	BEP × 1	DFS
11	0.4 years	F	Abdominal distension	469,73	Liver	No	JEB × 4	Total excision	Necrosis	JEB × 2	DFS
12	8.1 years	F	Difficulty voiding	40,300	Liver	No	JEB × 4	Total excision	MT	JEB × 2	DFS
13	1.0 year	F	Perianal abscess	11.8	No	Yes (IT)	No	Total excision	Grade II IT + EC	JEB × 4	Recurrence (YST, 1.4 years later), DFS
14	1.6 year	F	Buttock asymmetry	11,506	Liver, lung, bone, lymph node	No	JEB × 7	Total excision	MT	No	DFS
15	3.1 years	F	Buttock asymmetry	174,534	No	Yes (YST)	JEB × 4	Total excision	MT	JEB × 2	DFS

ACT = adjuvant chemotherapy; BEP = bleomycin, etoposide, and cisplatin; DFS = disease-free survival; EC = Embryonal carcinoma; IT = immature teratoma; JEB = carboplatin, etoposide, and bleomycin; MT = mature teratoma; YST = yolk sac tumor.

∗ The histology examination was done after neoadjuvant chemotherapy.

**Figure 1:**
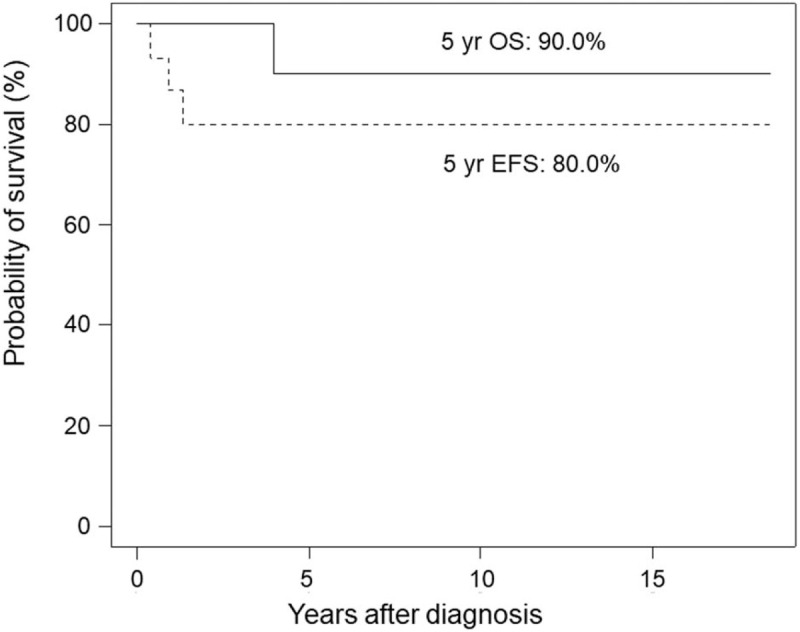
The 5-year overall survival and event-free survival of the 15 patients with malignant sacrococcygeal germ cell tumors.

The median follow-up period was 4.9 years (range, 2–18.4 years). Recurrence of MSGCTs occurred in 3 patients. One (case No. 1) was diagnosed with malignant recurrence with a yolk sac component (AFP level 330,181 ng/mL) at 2.1 years of age from a neonatal sacrococcygeal mature teratoma. Although salvage therapy was effective initially, repeated local recurrence and subsequent distant pulmonary metastasis occurred. The patient died of progressive disease 3.9 years after the diagnosis of MSGCT. The second patient (case No. 4) had malignant recurrence with the yolk sac component (AFP level 994.9 ng/mL) at 5 months of age from a neonatal sacrococcygeal grade II immature teratoma. The recurrent tumor was located in the posterior mediastinum with intraspinal extension. The patient underwent salvage therapy and remained disease-free and alive until the last follow-up. The third patient (case No. 13) was diagnosed with mixed germ cell tumor (grade II immature teratoma and embryonal carcinoma) after the neonatal period. The patient underwent tumor excision and received adjuvant chemotherapy. However, local recurrence was found 1.4 years later. The patient remained disease-free and alive after salvage therapy. The 5-year OS and EFS of the 15 patients with MSGCTs were 90% and 80%, respectively (Fig. 1).

## 4 Discussion

Sacrococcygeal germ cell tumors usually occur in patients in 2 clinical scenarios, based on the age of diagnosis. For those diagnosed in the neonatal period, the tumors usually present as a large exophytic mass around birth. Some sacrococcygeal tumors can be detected by prenatal sonography; the large tumor size can be measured prenatally and can affect the mode of delivery. In many cases, the best method of delivery for neonates with a sacrococcygeal germ cell tumor is the cesarean section to prevent labor dystocia, tumor rupture, and hemorrhage.^[8]^ In our study, 6 out of 7 patients who were diagnosed in the neonatal period were delivered by cesarean section. Only 1 patient was born by normal spontaneous delivery, and the tumor was not detected on prenatal ultrasonography.

Teratoma with or without yolk sac components is the predominant histology type in patients diagnosed in the neonatal period.^[1]^ Complete tumor excision with coccygectomy is the main treatment,^[9]^ and active surveillance without adjuvant chemotherapy has been recommended.^[10]^ We administered adjuvant chemotherapy carefully in 2 patients. One had grade III immature teratoma, and the other had grade II immature teratoma with a yolk sac component. Both patients did not develop recurrence. However, 1 patient with grade II immature teratoma who did not undergo adjuvant chemotherapy developed metastatic recurrence 1.4 years later. Salvage therapy with surgical excision and chemotherapy was effective. We were unable to compare the benefits of adjuvant chemotherapy in this setting due to the small number of cases.

The primary treatment of neonatal sacrococcygeal mature teratomas is early surgical resection with complete excision of the coccyx followed by serial serum AFP determinations to ensure the occurrence of physiological normalization of AFP levels and to facilitate early detection of tumor recurrence.^[11,12]^ Recurrence is seen in 2% to35% of patients with neonatal sacrococcygeal mature teratoma.^[13]^ It has been reported that almost 50% of recurrences after neonatal sacrococcygeal mature teratoma are malignant. The recurrent tumors usually contain secreting yolk sac components.^[9]^ Many reports suggested close follow-up for at least 3 years after surgery for neonatal sacrococcygeal teratomas, as most recurrences occurred within 3 years.^[14–16]^ Yao et al^[14]^ identified risk factors for any recurrence of neonatal sacrococcygeal teratomas after tumor resection including spillage of tumor parenchyma during operation, incomplete resection, and primary immature and malignant histology.^[14]^ However, Yoshida et al^[3]^ failed to find risk factors for malignant recurrence of neonatal sacrococcygeal teratomas after tumor resection. Salvage chemotherapy with BEP or JEB regimen would be effective for patients with malignant recurrence of neonatal sacrococcygeal mature teratoma.^[3,14,17]^ The true recurrence rate was unable to estimate in this study because we did not include all patients with neonatal sacrococcygeal mature teratoma. Three of the 7 patients with neonatal sacrococcygeal germ cell tumors in our cohort had recurrences at 1.4, 1.6, and 2.1 years, respectively. Two of them had recurrence with elevated AFP levels suggestive of the presence of yolk sac components. Only one tumor was operable after recurrence, and the yolk sac tumor was histologically confirmed. Our findings are consistent with previous studies.

Sacrococcygeal germ cell tumors can develop after the neonatal period, usually before 3 years of age. Different from the presence of an external palpable mass in the neonates, they tend to present as pain on sitting, buttock asymmetry, or bladder, bowel, or lower limb dysfunction. They are more likely to have malignant components.^[18]^ Unlike neonates with sacrococcygeal germ cell tumors, neoadjuvant chemotherapy followed by delayed tumor resection has been suggested for such cases.^[18]^ In our study, only one patient underwent primary tumor excision because the initial serum AFP was normal and the biopsy showed findings consistent with immature teratoma. The final pathologic diagnosis was mixed germ cell tumors (grade II immature teratoma and embryonal carcinoma). We also found that necrosis and mature teratoma were the common pathologic findings of the resected residual tumors after neoadjuvant chemotherapy as suggested in previous reports.^[19,20]^

An early report from the POG/CCG) trial showed a 4-year OS of 90% and EFS of 84% in 74 children with MSGCTs.^[7]^ In that trial, they adapted initial resection followed by 4 cycles of cisplatin-based chemotherapy for patients (40.8%) with an operable tumor. Patients (59.2%) whose tumor was unable to be removed completely initially received 4 cycles of cisplatin-based chemotherapy followed by delayed resection. In the same year, a German group reported a 5-year OS of 81% and EFS of 76% in 66 children with MSGCTs treated with German Cooperative Protocols MAKEI 83/86 and 89.^[18]^ The treatment strategy was similar to that in the POG/CCG trial. Delayed resection was performed after 4 cycles of cisplatin-based chemotherapy in 47% of patients. In total, 8 cycles and 6 cycles of cisplatin-based chemotherapy were applied in MAKEI 83/86 and 89, respectively. Recently, De Corti et al^[9]^ reported a good prognosis in 57 children with MSGCTs in France. The 5-year OS and EFS were 82% and 78%, respectively. They carried out risk-adapted chemotherapy followed by surgery. Low-risk patients did not require adjuvant therapy. Intermediate-risk patients received a vinblastine, bleomycin, and cisplatin regimen, and high-risk patients received a etoposide, ifosfamide, and cisplatin regimen. Although the number of cases in our study was small, the treatment outcomes were comparable to those in Western countries.

In conclusion, we presented the clinicopathological characteristics of MSGCTs in Taiwanese children. The treatment strategy was different between children with MSGCTs diagnosed in the neonatal period and those diagnosed after the neonatal period. Children with MSGCTs have a good overall prognosis. For those with sacrococcygeal mature teratoma or low-grade immature teratoma in the neonatal period, we recommend close follow-up for at least 3 years after surgery to detect malignant recurrence.

## Author contributions

**Conceptualization:** Shih-Hsiang Chen, Chia-Jui Du.

**Data curation:** Shih-Hsiang Chen, Chia-Jui Du, Tsung-Yen Chang.

**Formal analysis:** Shih-Hsiang Chen.

**Funding acquisition:** Shih-Hsiang Chen.

**Investigation:** Jin-Yao Lai, Yung-Ching Ming, Chuen Hsueh.

**Supervision:** Chao-Ping Yang, Iou-Jih Hung, Tang-Her Jaing.

**Validation:** Chao-Ping Yang, Iou-Jih Hung, Tang-Her Jaing.

**Visualization:** Chao-Ping Yang, Iou-Jih Hung, Tang-Her Jaing.

**Writing – original draft:** Shih-Hsiang Chen, Chia-Jui Du.

**Writing – review & editing:** Shih-Hsiang Chen.

## Abbreviations

Abbreviations: AFP = alpha-fetoprotein, BEP = cisplatin, etoposide, and bleomycin, EFS = event-free survival, JEB = carboplatin, etoposide, and bleomycin, MSGCT = malignant sacrococcygeal germ cell tumor, OS = overall survival.

## References

[1] Shaikh F Murray MJ Amatruda JF. Paediatric extracranial germ-cell tumours. Lancet Oncol 2016;17:e149–62. 27300675 10.1016/S1470-2045(15)00545-8

[2] Schneider DT Calaminus G Koch S. Epidemiologic analysis of 1,442 children and adolescents registered in the German germ cell tumor protocols. Pediatr Blood Cancer 2004;42:169–75. 14752882 10.1002/pbc.10321

[3] Yoshida M Matsuoka K Nakazawa A. Sacrococcygeal yolk sac tumor developing after teratoma: a clinicopathological study of pediatric sacrococcygeal germ cell tumors and a proposal of the pathogenesis of sacrococcygeal yolk sac tumors. J Pediatr Surg 2013;48:776–81. 23583133 10.1016/j.jpedsurg.2012.08.028

[4] Liu YL Lo WC Chiang CJ. Incidence of cancer in children aged 0-14 years in Taiwan, 1996-2010. Cancer Epidemiol 2015;39:21–8. 25599927 10.1016/j.canep.2014.11.010

[5] Hou JY Liu HC Yeh TC. Treatment results of extracranial malignant germ cell tumor with regimens of cisplatin, vinblastine, bleomycin or carboplatin, etoposide, and bleomycin with special emphasis on the sites of vagina and testis. Pediatr Neonatol 2015;56:301–6. 25769700 10.1016/j.pedneo.2014.12.003

[6] Kim J Lee NH Lee SH. Prognostic factors in children with extracranial germ cell tumors treated with cisplatin-based chemotherapy. Korean J Pediatr 2015;58:386–91. 26576183 10.3345/kjp.2015.58.10.386 PMC4644767

[7] Rescorla F Billmire D Stolar C. The effect of cisplatin dose and surgical resection in children with malignant germ cell tumors at the sacrococcygeal region: a pediatric intergroup trial (POG 9049/CCG 8882). J Pediatr Surg 2001;36:12–7. 11150431 10.1053/jpsu.2001.19993

[8] Chen SH. Neonatal solid tumors: a therapeutic challenge. Pediatr Neonatol 2018;59:1–2. 29339050 10.1016/j.pedneo.2018.01.010

[9] De Corti F Sarnacki S Patte C. Prognosis of malignant sacrococcygeal germ cell tumours according to their natural history and surgical management. Surg Oncol 2012;21:e31–7. 22459912 10.1016/j.suronc.2012.03.001

[10] Marina NM Cushing B Giller R. Complete surgical excision is effective treatment for children with immature teratomas with or without malignant elements: a Pediatric Oncology Group/Children's Cancer Group Intergroup Study. J Clin Oncol 1999;17:2137–43. 10561269 10.1200/JCO.1999.17.7.2137

[11] Huddart SN Mann JR Robinson K. Sacrococcygeal teratomas: the UK Children's Cancer Study Group's experience. I. Neonatal Pediatr Surg Int 2003;19:47–51. 12721723 10.1007/s00383-002-0884-2

[12] Egler RA Gosiengfiao Y Russell H. Is surgical resection and observation sufficient for stage I and II sacrococcygeal germ cell tumors? A case series and review. Pediatr Blood Cancer 2017;64:e26311. 10.1002/pbc.26311 27786428

[13] Padilla BE Vu L Lee H. Sacrococcygeal teratoma: late recurrence warrants long-term surveillance. Pediatr Surg Int 2017;33:1189–94. 28894920 10.1007/s00383-017-4132-1

[14] Yao W Li K Zheng S. Analysis of recurrence risks for sacrococcygeal teratoma in children. J Pediatr Surg 2014;49:1839–42. 25487496 10.1016/j.jpedsurg.2014.09.036

[15] Niramis R Anuntkosol M Buranakitjaroen V. Long-term outcomes of sacrococcygeal germ cell tumors in infancy and childhood. Surg Res Pract 2015;2015: Article ID: 398549. 10.1155/2015/398549 PMC4609489 26504900

[16] Sinha S Kumar Sarin YVPD. Neonatal sacrococcygeal teratoma: our experience with 10 cases. J Neonatal Surg 2013;2:eCollection 4. PMC4420341 26023424

[17] Mann JR Gray ES Thornton C. Mature and immature extracranial teratomas in children: the UK Children's Cancer Study Group Experience. J Clin Oncol 2008;26:3590–7. 18541896 10.1200/JCO.2008.16.0622

[18] Gobel U Schneider DT Calaminus G. Multimodal treatment of malignant sacrococcygeal germ cell tumors: a prospective analysis of 66 patients of the German cooperative protocols MAKEI 83/86 and 89. J Clin Oncol 2001;19:1943–50. 11283126 10.1200/JCO.2001.19.7.1943

[19] Daneshmand S Djaladat H Nichols C. Management of residual mass in nonseminomatous germ cell tumors following chemotherapy. Ther Adv Urol 2011;3:163–71. 21969846 10.1177/1756287211418721 PMC3175700

[20] Heerema-McKenney A Bowen J Hill DA. Members of Cancer Committee CoAP. Protocol for the examination of specimens from pediatric and adult patients with extragonadal germ cell tumors. Arch Pathol Lab Med 2011;135:630–9. 21526961 10.5858/2010-0405-CP.1

